# Mapping and Identification of Antifungal Peptides in the Putative Antifungal Protein AfpB from the Filamentous Fungus *Penicillium digitatum*

**DOI:** 10.3389/fmicb.2017.00592

**Published:** 2017-04-06

**Authors:** Sandra Garrigues, Mónica Gandía, Attila Borics, Florentine Marx, Paloma Manzanares, Jose F. Marcos

**Affiliations:** ^1^Department of Food Biotechnology, Instituto de Agroquímica y Tecnología de Alimentos, Consejo Superior de Investigaciones CientíficasValencia, Spain; ^2^Institute of Biochemistry, Biological Research Centre of Hungarian Academy of SciencesSzeged, Hungary; ^3^Division of Molecular Biology, Biocenter, Medical University of InnsbruckInnsbruck, Austria

**Keywords:** antimicrobial peptides, antifungal proteins, protein mapping, peptide design, *Penicillium digitatum*, postharvest pathology, *Penicillium chrysogenum*, synergy

## Abstract

Antifungal proteins (AFPs) from Ascomycetes are small cysteine-rich proteins that are abundantly secreted and show antifungal activity against non-producer fungi. A gene coding for a class B AFP (AfpB) was previously identified in the genome of the plant pathogen *Penicillium digitatum*. However, previous attempts to detect the AfpB protein were not successful despite the high expression of the corresponding *afpB* gene. In this work, the structure of the putative AfpB was modeled. Based on this model, four synthetic cysteine-containing peptides, PAF109, PAF112, PAF118, and PAF119, were designed and their antimicrobial activity was tested and characterized. PAF109 that corresponds to the γ-core motif present in defensin-like antimicrobial proteins did not show antimicrobial activity. On the contrary, PAF112 and PAF118, which are cationic peptides derived from two surface-exposed loops in AfpB, showed moderate antifungal activity against *P. digitatum* and other filamentous fungi. It was also confirmed that cyclization through a disulfide bridge prevented peptide degradation. PAF116, which is a peptide analogous to PAF112 but derived from the *Penicillium chrysogenum* antifungal protein PAF, showed activity against *P. digitatum* similar to PAF112, but was less active than the native PAF protein. The two AfpB-derived antifungal peptides PAF112 and PAF118 showed positive synergistic interaction when combined against *P. digitatum*. Furthermore, the synthetic hexapeptide PAF26 previously described in our laboratory also exhibited synergistic interaction with the peptides PAF112, PAF118, and PAF116, as well as with the PAF protein. This study is an important contribution to the mapping of antifungal motifs within the AfpB and other AFPs, and opens up new strategies for the rational design and application of antifungal peptides and proteins.

## Introduction

There is an urgent need to develop new antifungal molecules with properties and mechanisms of action different from existing ones (Brown et al., 2012; Fisher et al., 2012). Antimicrobial peptides and proteins (AMPs) have been found in a broad variety of species (Hancock and Sahl, 2006) and are candidates for the development of novel therapeutic compounds (Zasloff, 2002; Brogden, 2005; Fjell et al., 2012) including antifungals (Shah and Read, 2013). A remarkable group of AMPs are the antifungal proteins (AFPs) of fungal origin. AFPs belong to the broad class of defensins and are produced by some species of filamentous Ascomycetes, mostly from the genus *Aspergillus* and *Penicillium* (Marx et al., 2008; Meyer, 2008; Galgóczy et al., 2010; Silva et al., 2014). AFPs are small (∼50 amino acid residues), secreted, amphipathic, cationic and cysteine-rich proteins (CRPs) that contain six to eight cysteine residues and fold into compact disulfide-stabilized structures with five β-strands, which confer high stability under adverse biochemical and biophysical conditions (Batta et al., 2009). All the AFPs contain the so-called γ-core, a three-dimensional peptide signature present in antimicrobial CRPs spanning biological kingdoms (Yount and Yeaman, 2004). One of the most studied AFPs is the *Penicillium chrysogenum* PAF protein (55 amino acids in length) that can be easily purified from the culture supernatants of the producer fungus *P. chrysogenum* (Marx et al., 2008). PAF inhibits the growth of filamentous human and plant pathogenic fungi at μM concentrations and is non-toxic to mammalian cells *in vitro* (Marx et al., 2008) and *in vivo* (Palicz et al., 2013).

A significant diversity of AFP-like genes and proteins are widespread in Ascomycetes, with genomes that encode up to three different sequence-related AFPs (Galgóczy et al., 2013; Garrigues et al., 2016; Tóth et al., 2016), providing a rich source of potentially divergent antifungals. Bioinformatic and phylogenetic analyses suggested the classification of the different AFP-like sequences in at least three different classes: A, B, and C (Garrigues et al., 2016). Notably, these fungal genomes code for a different number of AFP-like proteins that belong to diverse classes; *P. chrysogenum* encodes three classes (A, B, and C), *Penicillium roqueforti* two classes (A and C), and *Penicillium digitatum* just one (B). There is also a significant variation in the amount of AFP produced by each fungus and the growth conditions required to achieve maximum yields. Thus, the above-mentioned PAF protein is secreted in large amounts by *P. chrysogenum* (Marx et al., 2008), while the NFAP and NFAP2 from *Neosartorya fischeri* are produced in modest amounts (Kovács et al., 2011; Virágh et al., 2014). An extreme example of this scenario is that of the previously characterized *afpB* gene identified in the genome of the phytopathogenic fungus *P. digitatum* (Garrigues et al., 2016). Attempts to identify the class B AFP (AfpB) in the small size protein fraction secreted by the fungus were not successful, even in the constitutive expressing strains that produce up to 1,000 times more *afpB* mRNA than the wild-type (Garrigues et al., 2016). This unexpected finding was one of the main reasons that led us to conduct the study reported here.

In-depth understanding of the structure and mode of action of AFPs is required for their potential future application as antifungal compounds. In addition, AFP and AFP-like sequences are a rich source for the identification and rational design of novel antimicrobial peptides with improved properties (Marcos et al., 2008, 2012). Finally, the structural domains that account for the antifungal activity of AFPs remain to be fully identified and characterized. In this study, the structure of the putative *P. digitatum* AfpB protein was predicted by *in silico* molecular modeling to aid in the design of peptides based on the AfpB primary sequence and structure, whose antifungal activity was demonstrated and characterized. With this rational approach, peptides that have moderate but specific antifungal activity were identified within the AfpB and PAF amino acid sequences. Importantly, the previously characterized PAF26 hexapeptide (Muñoz et al., 2013a) showed positive synergistic interaction with the AFP-derived peptides and the PAF protein.

## Materials and Methods

### Strains and Media

The fungal strains used in this study are *P. digitatum* CECT20796, *P. chrysogenum* Q176, *Botrytis cinerea* CECT2100, *Fusarium oxysporum* 4287, and *Aspergillus niger* CBS 120.49. Fungi were cultured on potato dextrose agar (Difco-BD Diagnostics, Sparks, MD, USA) plates for 7–10 days at 24°C with the exception of *F. oxysporum*, which was cultured on potato dextrose broth (PDB; Difco-BD Diagnostics) at 28°C for 4 days with shaking. Conidia (mitotic asexual spores) were collected, filtered, and adjusted to the appropriate concentration. For antibacterial assays, the Gram-negative bacterium *Escherichia coli* JM109 was grown in Luria-Bertani (LB) medium at 37°C with shaking.

### *In silico* Calculations and AfpB Structure Prediction

The SWISS-MODEL program^1^ (Arnold et al., 2006) was used to predict the 3D structure of the *P. digitatum* antifungal protein AfpB (Garrigues et al., 2016) using the *P. chrysogenum* antifungal protein PAF as template (PDB ID 2MHV; Fizil et al., 2015). The model obtained was refined using the ModRefiner tool^2^ (Xu and Zhang, 2011) and subsequently validated by RAMPAGE^3^ to ensure that all the amino acids of the AfpB model were located inside the favored and energetically allowed regions according to the Ramachandran plot (Lovell et al., 2003).

The theoretical molecular mass, the pI, and the GRAVY of the mature AfpB protein and peptides derived therefrom were examined with the Compute pI/Mw and ProtParam tools of the ExPASy Proteomics Server^4^ (Gasteiger et al., 2005), respectively. The signal peptide and disulfide bridges of the mature protein were predicted using the SignalP 4.0 Server^5^ (Petersen et al., 2011) and the DISULFIND software^6^ (Ceroni et al., 2006), respectively. The 3D model of AfpB was visualized by Chimera software (Pettersen et al., 2004).

### PAF Protein and Synthetic Peptides

The PAF protein was produced by *P. chrysogenum* and purified as previously described (Batta et al., 2009; Sonderegger et al., 2016). All the peptides used in this work were synthetic and purchased at >95% purity from GenScript (Piscataway, NJ, USA) wherein they were synthesized by solid phase methods using *N*-(9-fluorenyl) methoxycarbonyl (Fmoc) chemistry. The sequences of the peptides used and their physicochemical properties are listed in **Table 1**. Peptides PAF109, PAF112, PAF118, and PAF119 derived from the AfpB protein, and peptide PAF116 derived from the PAF protein, were synthetized with a disulfide bond linking the two cysteine residues, respectively. Stock solutions of peptides were prepared at 5.12 mM in 10 mM 3-(*N*-morpholino)-propanesulfonic acid (MOPS; Sigma-Aldrich, St. Louis, MO, USA) pH 7 and stored at -20°C. The peptide concentrations were prepared by dissolving a given peptide amount (mg) in MOPS buffer (mL), considering the purity of each of the peptides provided by the manufacturer. Additionally, the PAF26 concentration was confirmed spectrophotometrically by measuring the absorbance at 280 nm (𝜀280 = 5600 M^-1^ cm^-1^ for W residue).

**Table 1:** Amino acid sequences and properties of the peptides used in this study.

ID	Sequence^1^	MM (Da)	Net charge	pI	GRAVY	Source
PAF26	RKKWFW	950.2	3.0	11.2	-1.883	López-García et al., 2002
PAF109	GQCSLKHNTCT	1189.3	1.1	8.1	-0.718	AfpB (*P. digitatum*)
PAF112	NCGSAANKRAKSDRHHCE	1982.1	2.2	8.9	-1.600	AfpB (*P. digitatum*)
PAF113	NAGSAANKRAKSDRHHAE	1920.0	2.2	10.0	-1.678	AfpB (*P. digitatum*)
PAF118	NTCTYLKGGRNVIVNCG	1810.1	2.0	8.9	-0.065	AfpB (*P. digitatum*)
PAF119	HCEYDEHHRRVDCQ	1824.9	-1.7	5.8	-2.014	AfpB (*P. digitatum*)
PAF116	KCPKFDNKKATKDNNKCT	2081.4	4.0	9.5	-1.906	PAF (*P. chrysogenum*)

*^1^Underlined cysteine residues in PAF109, PAF112, PAF116, PAF118, and PAF119 are linked by disulfide bonds.*

### ECD Spectroscopy

Electronic circular dichroism (ECD) spectroscopic measurements were performed in the 195–260 nm wavelength range (far-UV) to determine the secondary structure of the AfpB-derived peptides. Peptide samples were dissolved in H_2_O, and in a 50:50 trifluoroethanol (TFE):H_2_O mixture at approximately 0.1 mg/mL concentration and measured in a 0.1 cm path-length quartz cuvette using a Jasco J-815 spectropolarimeter at a scan speed of 100 nm/s at 25°C. Solvent spectra were measured similarly and subtracted from the corresponding spectra of peptides. Ellipticity data were given in mdeg units.

### Antimicrobial Activity Assays

Growth inhibition assays were performed in 96-well microtiter plates (Nunc, Roskilde, Denmark) in a total volume of 100 μL as described previously (López-García et al., 2002). A volume of 90 μL of fungal conidia (2.5 × 10^4^ conidia/mL) or bacterial cells (5 × 10^5^ cells/mL) in appropriate growth media [1/20 diluted PDB containing 0.01% (w/v) chloramphenicol for fungi or 1/10 diluted LB for bacteria] was mixed in each well with 10 μL of 10× concentrated peptide or PAF protein solution from serial twofold dilutions (from 1 to 128 μM for PAF109, PAF112, PAF113, PAF116, PAF118, and PAF119; from 0.25 to 4 μM for PAF26; and from 1 to 16 μM for the protein PAF from *P. chrysogenum*). All samples were prepared in triplicate. Plates were statically incubated for 72 h at the optimal temperature of each microorganism. Growth was determined by measuring the optical density (OD) at 600 nm (OD_600_) using a Multiskan Spectrum plate spectrophotometer (Thermo Electron Corporation, Vantaa, Finland), and the OD_600_ mean and SD were calculated. Dose–response curves were generated from measurements after 72 h for fungi, and 48 h for *E. coli*. These experiments were repeated at least twice. The minimum inhibitory concentration (MIC) is the peptide concentration that completely inhibited growth in all the experiments performed.

For assays of synergy, different combinations of peptides and concentrations were tested in 96-well microtiter plates. Ten microliters of two different 10× concentrated peptides were mixed in the same well with 80 μL of *P. digitatum* conidia prepared and grown as above (total volume 100 μL). Plates were statically incubated for 72 h at 24°C. Data are expressed as OD_600_ mean ± SD of three replicates. Sub-MICs for each peptide were: 1–32 μM for PAF112, 1–16 μM for PAF118, 1–8 μM for PAF116, and 0.25–2 μM for PAF26. Similarly, the interaction between the PAF protein from *P. chrysogenum* and the PAF26 peptide was tested against *A. niger*, *P. chrysogenum*, and *P. digitatum*.

### Proteolytic Digestion Assays

The proteolytic digestion assays were performed as described (Ferre et al., 2006; López-García et al., 2015) with minor modifications. Peptides (5 μM) were dissolved in 10 mM MOPS pH 7 and digested with 5 μg/mL of recombinant proteinase K (2 U/mg; Roche, Mannheim, Germany) at 30°C. Aliquots were withdrawn from the reaction mixtures at 0, 1, 2, and 24 h of incubation and immediately heated at 80°C for 10 min to inactivate the enzyme. Two replicates were prepared for each treatment, and the experiments were repeated at least twice.

Analyses of peptide digests were conducted by RP-HPLC using a Waters system (Waters Corporation, Milford, MA, USA) equipped with a 1525 Binary HPLC pump, a 2996 Photodiode Array Detector, and a 717 plus Autosampler. A Symmetry C18 column (4.6 mm × 150 mm, 5 μm, Waters Corporation) kept at 40°C was operated at a flow rate of 1 mL/min. Peptides were eluted with a linear gradient of solvent B [acetonitrile with 0.1% trifluoroacetic acid (TFA)] in solvent A (water with 0.1% TFA) 0–40% in 20 min and detected at 214 nm.

### Statistical Analysis

All the statistical analyses carried out to determine the synergistic interactions were performed using STATGRAPHICS Centurion 16.1.17^7^. The significant differences between sets of data were determined by a bi-factorial ANOVA and Tukey’s honestly significant difference (HSD) test. Significance was regarded as *p* < 0.01.

## Results

### AfpB Structural Modeling

**FIGURE 1:**
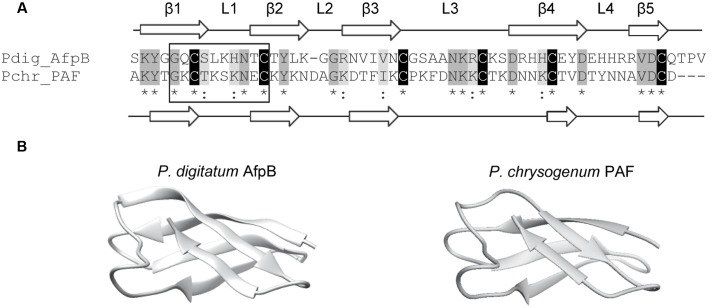
**Molecular modeling of the *P. digitatum* class B antifungal protein AfpB. (A)** Amino acid sequence alignment of AfpB and *P. chrysogenum* antifungal protein PAF. Cysteines are shadowed in black, other conserved amino acids in dark gray, and similar amino acids in light gray. Asterisks below sequences indicate conserved amino acids and colons similar amino acids. PAF and predicted AfpB β-sheets are labeled as β1 to β5 white arrows; and loops are labeled as L1 to L4. The γ-core motif is boxed. **(B)** Comparison of the tertiary structure of PAF (right) with the 3D molecular model obtained for AfpB (left).

**FIGURE 2:**
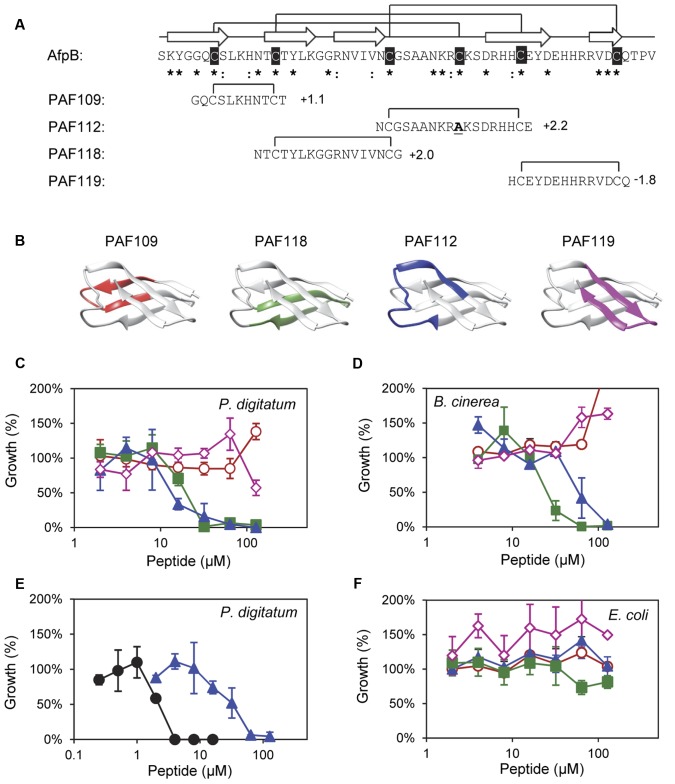
**Design and *in vitro* inhibitory activity of the synthetic AfpB-derived peptides. (A)** Amino acid sequence and net charge of the AfpB-derived peptides and their location in the protein sequence. Cysteine residues are shadowed in black. Asterisks and colon show conserved residues (see **Figure 1**). Arrows show the predicted β-sheets in AfpB. Black lines show the predicted disulfide bond pattern for AfpB (top) and the disulfide bonds in the AfpB-derived PAF109, PAF112, PAF118, and PAF119 (bottom). **(B)** Color-coded location of the AfpB-derived peptides in the modeled tertiary structure of AfpB. **(C–F)** Color-coded dose–response curves showing the antimicrobial activity of the peptides PAF109 (red circles), PAF118 (green squares), PAF112 (blue triangles), PAF119 (pink diamonds), and PAF26 (black circles), tested against *P. digitatum* **(C,E)**, *B. cinerea* **(D)**, and *E. coli* **(F)**. Dose–response curves show mean ± SD percentage of control growth of triplicate samples after 72 h of incubation at 24°C for fungi **(C–E)** and 48 h at 37°C for bacteria **(F)**.

*In silico* translation of the *afpB* gene (PDIG_68840) shows that the AfpB primary structure contains 92 amino acid residues (Garrigues et al., 2016). A signal peptide and a pro-peptide (35 residues in total) are predicted to be cleaved from the N-terminal end of the protein, liberating the mature AfpB which is a small basic CRP consisting of 57 amino acid residues (**Figure 1A**). It has a calculated molecular mass of 6.46 kDa, a pI value of 9.06 and a GRAVY of -1.084. The dextromeric isoform of the γ-core with the consensus sequence GXCX_3-9_C (Yount and Yeaman, 2004) is present near the N-terminus (**Figure 1A**, boxed). The structure of mature AfpB was predicted by homology modeling using the SWISS-MODEL online resource, which selected the protein PAF from *P. chrysogenum* as template for prediction (PDB ID 2MHV; Fizil et al., 2015), and refined using the ModRefiner tool (**Figure 1**). AfpB shows 33% amino acid identity and 44% similarity with the *P. chrysogenum* PAF. The refined AfpB model showed 56 of 57 (98%) of the amino acid residues positioned in energetically favored regions and one residue in energetically allowed regions according to the Ramachandran plot (Supplementary Figure S1). The structural model of AfpB contains five antiparallel β-strands connected by three small loops and a big surface-exposed loop (Supplementary Figure S2 and **Figure 1**). The antiparallel β-strands create two packed β-sheets and the six conserved cysteine residues form three disulfide bridges. The most probable disulfide bond pattern predicted by the DISULFIND software is “abcabc,” between cysteines 7 and 35; 14 and 42; and 27 and 53 (see also **Figure 2A**), which also corresponds to the disulfide bond pattern determined in PAF (Váradi et al., 2013).

### Identification of AfpB-Derived Antifungal Peptides with Specific Antifungal Activity

To identify putative antimicrobial motifs in AfpB, the cysteine-containing peptides PAF109, PAF112, PAF118, and PAF119 were derived from the AfpB sequence (**Table 1**). Peptides (**Figure 2A**) were rationally designed with intramolecular disulfide bonds to promote folding into a loop or β-hairpin structure corresponding to the regions within the protein (**Figure 2B**). Peptide PAF109 is cationic, spans the first loop L1 between β-sheets β1 and β2, and corresponds to the conserved γ-core motif. PAF118, also with a positive net charge, includes the β2 and β3 sheets linked by a small loop (L2). PAF112 extents over the largest surface-exposed cationic loop L3 of the protein located between β3 and β4. In this peptide, the central Cys35 in L3 has been replaced by an alanine in order to promote disulfide bond formation between the terminal cysteine residues (**Figure 2A**). An additional peptide derived from this largest surface-exposed cationic loop L3, called PAF113, was designed. PAF113 corresponds to the linear version of the peptide PAF112 in which all cysteine residues were substituted by alanines. Finally, PAF119 has a negative net charge as opposed to the other three peptides, and includes the last two β-sheets (β4 and β5) linked by the small C-terminal loop (L4).

**FIGURE 3:**
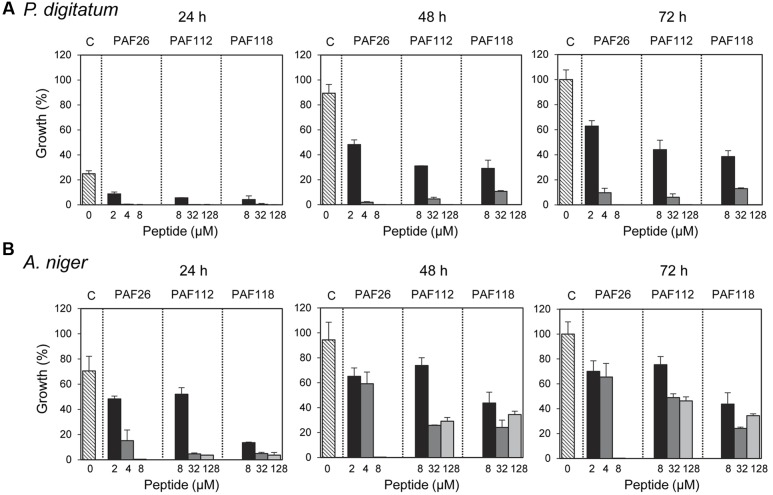
**Time-course inhibition of growth of filamentous fungi by synthetic peptides.** Time-course inhibition of growth of *P. digitatum* **(A)** and *A. niger* **(B)** in the presence of increasing concentrations of the synthetic peptides PAF26 (2, 4, and 8 μM), PAF112 (8, 32, and 128 μM), and PAF118 (8, 32, and 128 μM). Data are shown after 24, 48, and 72 h of incubation as indicated. Bars represent the mean value ± SD of the percentage of growth as compared to the 100% control that was defined for each fungus as the growth in the absence of peptide (C, control) at 72 h.

These AfpB-derived peptides were tested toward a selection of filamentous fungi that include plant pathogens (the citrus fruit-specific *P. digitatum*, the polyphagous *B. cinerea*, and the vascular wilt pathogen *F. oxysporum*), the PAF producer *P. chrysogenum*, and a strain from *A. niger* particularly sensitive to the PAF protein. The cationic peptides PAF112 and PAF118 showed antifungal activity and were completely inhibitory to *P. digitatum* and *B. cinerea*, with MIC values between 64 and 128 μM (**Figures 2C,D** and **Table 2**). At lower concentrations (8–32 μM), PAF112 and PAF118 showed partial inhibition of the growth of these two fungi (**Figure 3** and data not shown). In the case of *A. niger* and *P. chrysogenum*, the PAF112 and PAF118 peptides significantly delayed fungal growth (**Figure 3** and data not shown). Even at low concentrations (8–32 μM) an inhibition was observed at short incubation times (24 h), although the peptides were not completely inhibitory at the highest concentration tested (128 μM) (**Figure 3**). In some experiments, PAF118 showed slightly higher antifungal activity than PAF112 according to the dose–response effect (see **Figures 2**, **3**).

**Table 2:** Minimum inhibitory concentration (MIC) of peptides studied in this work^1^.

Microorganism	PAF26	PAF109	PAF112	PAF113	PAF118	PAF119	PAF116
*P. digitatum* CECT20796	8	NI^2^	64	64	64	NI	32
*B. cinerea* CECT2100	8	NI	128	128	64	NI	16
*F. oxysporum* 4287	16	NI	NI	NI	NI	NI	NI
*A. niger* CBS 120.49	8	NI	>128	>128	>128	NI	–^3^
*P. chrysogenum* Q176	16	NI	>128	>128	>128	NI	–
*E. coli* JM109	NI	NI	NI	NI	NI	NI	–

*^1^MIC values are shown in μM units for each fungus–peptide combination.*

*^2^NI, no significant inhibition at the highest concentration tested (128 μM).*

*^3^–, not tested.*

Regarding other peptides (**Figure 2**), PAF109 derived from the conserved γ-core did not show inhibitory activity at any of the concentrations tested. The anionic peptide PAF119 did not show activity either. None of the AfpB-derived peptides tested showed inhibitory effect against *F. oxysporum* at the maximum concentration used. This strain of *F. oxysporum* has shown comparatively higher tolerance to most of the peptides studied in our laboratory over the years (López-García et al., 2015).

The synthetic hexapeptide PAF26 is an antifungal peptide that was identified by a combinatorial approach against *P. digitatum* (López-García et al., 2002). PAF26 is a cationic tryptophan-rich peptide (**Table 1**) that belongs to the cell-penetrating class of AMPs and whose mechanism of action has been characterized previously (Muñoz et al., 2013a). PAF26 has specific activity for filamentous fungi showing MIC values below 10 μM and was therefore chosen as an internal control in this study. We found that peptides PAF112 and PAF118 are around one order of magnitude less active than PAF26 (**Figure 2E** and **Table 2**). As occurs with PAF26, none of the AfpB-derived peptides showed antibacterial activity against *E. coli* (**Figure 2F**), which demonstrated the selective antifungal activity of PAF112 and PAF118 over the range of concentrations assayed.

### Peptide PAF116 Derived from the *P. chrysogenum* Antifungal Protein PAF is Less Active than the Native Protein

**FIGURE 4:**
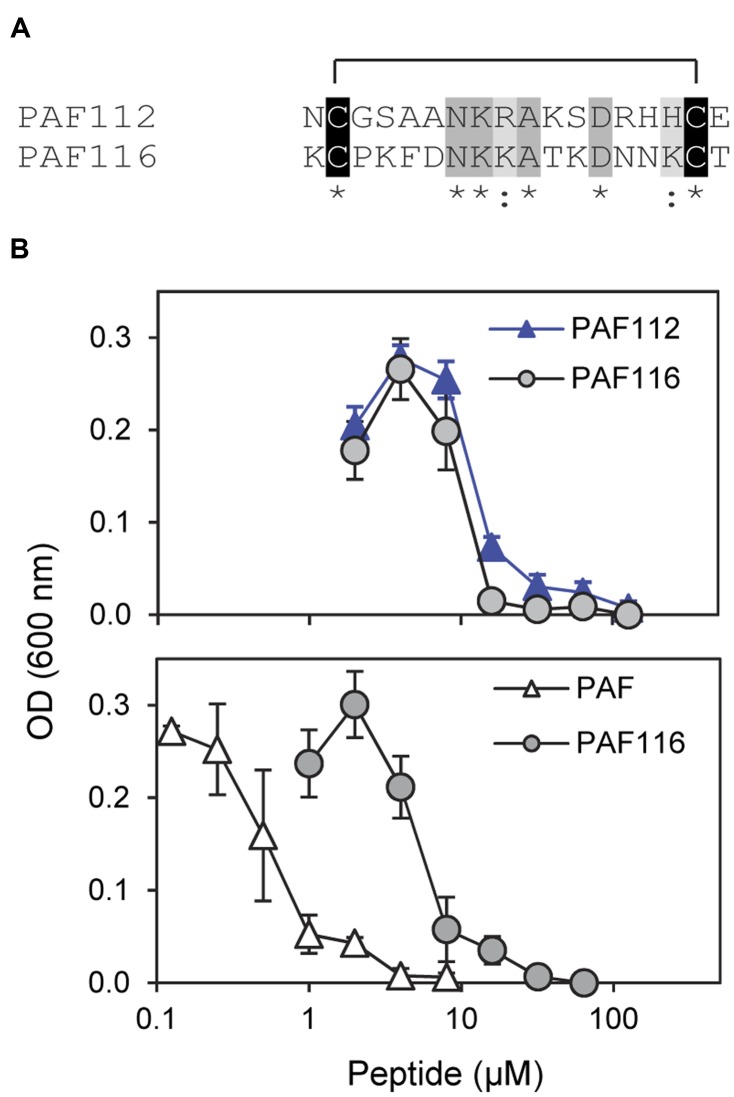
**Comparative study of the AfpB-derived peptide PAF112 and the PAF-derived PAF116. (A)** Amino acid sequence alignment of PAF112 and PAF116. Cysteine conserved residues are shadowed in black, conserved residues in dark gray, and similar in light gray. Asterisks and colon show conserved residues (see **Figure 1**). **(B)** Dose–response curves of *P. digitatum* inhibition by the synthetic peptides PAF112 (blue triangles) and PAF116 (gray circles) (top), and by the peptide PAF116 (gray circles) and protein PAF from *P. chrysogenum* (white triangles) (bottom). Curves show mean ± SD OD_600_ of triplicate samples after 72 h of incubation at 24°C.

Since it was impossible to detect AfpB in all the experiments conducted so far in our laboratory (Garrigues et al., 2016), we wanted to test whether peptides derived from AFPs could have antifungal activity similar to the native protein. Therefore, we designed the disulfide cyclized peptide PAF116 analogous to PAF112 but originating from the *P. chrysogenum* antifungal protein PAF, which was readily available (**Figure 4**). The sequence alignment of PAF112 and PAF116 showed low similarity (44%; **Figure 4A**). However, despite the sequence divergence between these two peptides, both shared comparable antifungal activity against *P. digitatum* (**Figure 4B**, top). As in the case of PAF112, PAF116 showed no antifungal activity against *F. oxysporum* (data not shown). In contrast, PAF116 exhibited higher antimicrobial activity than PAF112 against *B. cinerea* (**Table 2**).

Additional assays were conducted to compare the antifungal activity of the peptide PAF116 and the parental protein PAF (**Figure 4B**, bottom). The results showed that PAF116 is less active than the PAF protein against *P. digitatum*. The MIC values were 32 μM for the peptide PAF116 and 8 μM for the PAF protein. This result leads us to propose that AfpB would be also more active than the peptides PAF112 or PAF118 derived from its primary sequence. In addition, it is demonstrated that *P. digitatum* is sensitive to the PAF protein from *P. chrysogenum*, which as the producer fungus is tolerant to this protein (**Table 3**).

**Table 3:** Synergistic interaction between PAF26 peptide and PAF protein.

Strain	PAF26 peptide^1^	PAF protein^1^	Interaction PAF26/PAF
*P. digitatum* CECT20796	8	8	Positive synergy (*p* < 0.01)
*P. chrysogenum* Q176	16	NI^2^	No synergy
*A. niger* CBS 120.49	8	0.08	Positive synergy (*p* < 0.01)

*^1^MIC (μM) are shown for each combination.*

*^2^NI, no inhibition at the highest concentration tested (64 μM).*

### Synergistic Interactions

**FIGURE 5:**
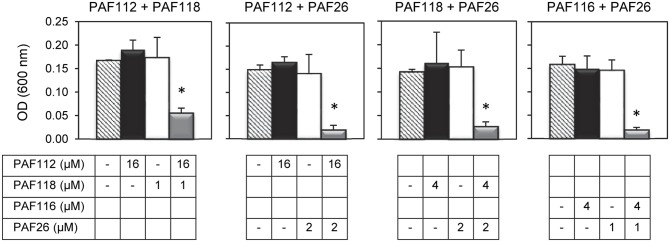
**Synergistic interactions between AFP-derived peptides and PAF26.** Each of the four panels show the synergistic interactions between PAF112 and PAF118; PAF112 and PAF26; PAF118 and PAF26; and PAF116 and PAF26, tested against *P. digitatum*. The graphics show mean ± SD OD_600_ values of triplicate samples after 48 h of incubation at 24°C. Asterisks show statistical significance for positive interaction (multifactorial ANOVA *p* < 0.01) of fungal growth inhibition for each peptide combination at the concentrations indicated below the panels.

In view of the above data we hypothesized that the combined action of different AfpB-derived peptides could mimic the activity of the native protein. To address our prediction, we designed and conducted a series of experiments in which different peptides were tested for synergy against *P. digitatum* (**Figure 5**). From all possible combinations of the four AfpB-derived peptides in groups of two, only PAF112 and PAF118 showed positive synergistic interaction when combined (**Figure 5** and data not shown). A multifactorial ANOVA test was conducted at different concentrations of PAF112 (from 1 to 32 μM), PAF118 (from 1 to 16 μM) and all possible concentration combinations of both, demonstrating the existence of an interaction (*p* < 0.01). As example, the data from the combined action of 16 μM PAF112 and 1 μM PAF118 is shown (**Figure 5**, left). Single treatments of PAF112 or PAF118 did not result in a fungal growth significantly different from the control. However, the combined activity of both peptides resulted in significant inhibition of *P. digitatum* (*p* < 0.01, **Figure 5**), in the range of the inhibition achieved by the PAF protein at 1 μM (**Figure 4B**). This observation leads us to suggest that these two motifs contribute to reach optimal activity of the native AfpB protein.

To determine the possible interaction between the hexapeptide PAF26 and the antifungal peptides derived from the AfpB and PAF protein, additional combinations of peptides were tested. These experiments demonstrated that the AfpB-derived peptides PAF112 and PAF118, as well as the PAF-derived peptide PAF116, show a positive synergistic interaction with PAF26 (multifactorial ANOVA, *p* < 0.01; **Figure 5**), reducing the fungal biomass when combined at non-inhibitory concentrations.

**FIGURE 6:**
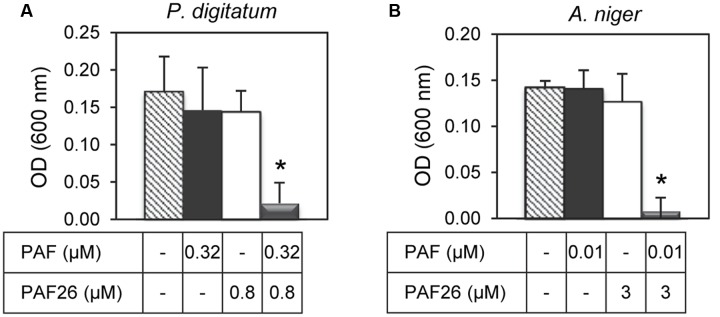
**Synergistic interactions between the protein PAF from *P. chrysogenum* and the hexapeptide PAF26.** The synergistic interaction was tested against *P. digitatum* **(A)** and *A. niger* **(B)**. The graphics show mean ± SD OD_600_ values of triplicate samples after 48 h of incubation at 24°C. Asterisks show statistical significance for positive interaction (multifactorial ANOVA *p* < 0.01) of fungal growth inhibition for each peptide/protein combinations at the concentrations indicated below the panels.

Given the synergy existing between PAF26 and the antifungal peptides derived from both AFPs, further antifungal assays were conducted to determine whether the protein PAF from *P. chrysogenum* could also interact synergistically with PAF26. Three fungi were chosen for these experiments that are representative of the sensitivity range to PAF, from the highly sensitive *A. niger* to the tolerant PAF-producer *P. chrysogenum* (**Table 3**). The results indicated no apparent synergy in assays with *P. chrysogenum*. For *P. digitatum* and the highly PAF-sensitive *A. niger*, PAF protein showed a positive synergistic interaction with the peptide PAF26 (**Figure 6** and **Table 3**). Interestingly, the three fungi did not show major differences in their sensitivity to PAF26 while they did to the PAF protein (**Table 3**), suggesting that the PAF protein and the peptide PAF26 could have differences in their mechanism of action.

### ECD Spectroscopic Analysis

**FIGURE 7:**
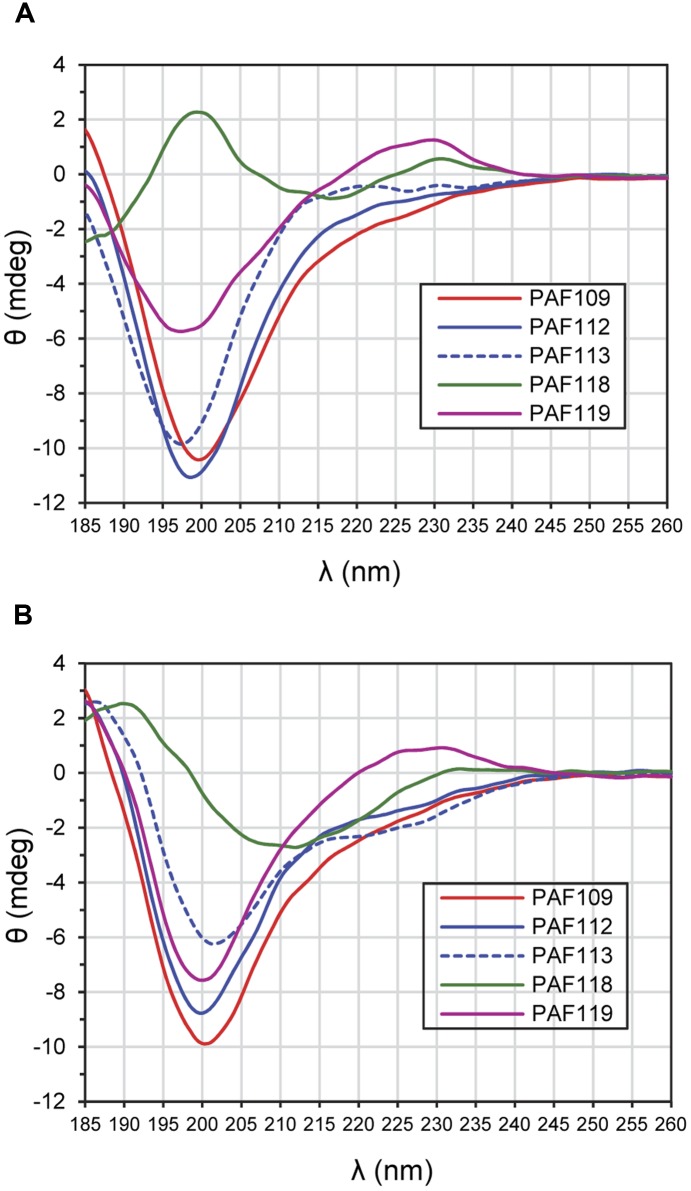
**ECD spectra of AfpB-derived peptides. (A)** Spectra obtained from peptides in H_2_O. **(B)** Spectra obtained from peptides solved in TFE/H_2_O (50:50) mixture. All experiments were conducted at 25°C.

Electronic circular dichroism spectroscopy is a sensitive tool for the study of the secondary structure of proteins and peptides. To investigate the structure of the AfpB-derived peptides, ECD spectroscopy was performed (**Figure 7**). ECD analyses revealed that peptides PAF109, PAF112, and PAF113 predominantly displayed unordered structure in water characterized by strong minima at 195–200 nm (**Figure 7A**). TFE is an organic solvent commonly used to induce intramolecular H-bonding, therefore inducing and stabilizing secondary structure of protein fragments in the absence of cooperatively stabilizing structural regions of the protein of origin. Upon addition of TFE, a minor helical contribution appeared in case of the linear peptide PAF113, reflected by a low intensity broad negative shoulder in the 220–225 nm region (**Figure 7B**). Since this peptide has no disulfide bond and therefore less conformational restraints, it can adapt more readily to changes in environment (for example, addition of a helix-inducing solvent). PAF119 (**Figure 7A**) yielded a spectrum that reports on the dominance of random structures with a minor β-sheet contribution, and a more pronounced signal from the disulfide bridge (maximum around 230 nm). Addition of TFE to PAF119 did not increase, but rather diminished β-sheet contributions.

PAF118 displayed markedly different spectra compared to those of the rest of the peptides analyzed (**Figure 7A**, green line). The ECD spectrum of PAF118 is dominated by contributions from antiparallel β-structure (relative maximum near 195 nm and minimum near 215 nm) and disulfide bonds (maximum at 230 nm). Addition of TFE increased the β-character of the spectrum while the presence of a positive band at around 230 nm indicative of disulfide bonds was maintained (**Figure 7B**). Therefore, PAF118 represents an antifungal motif from AfpB that folds by itself in an antiparallel β-structure, consistent with the structural prediction within the context of the protein (see also **Figure 2B**).

### AfpB-Derived Peptides Show Resistance to Proteolytic Degradation

**FIGURE 8:**
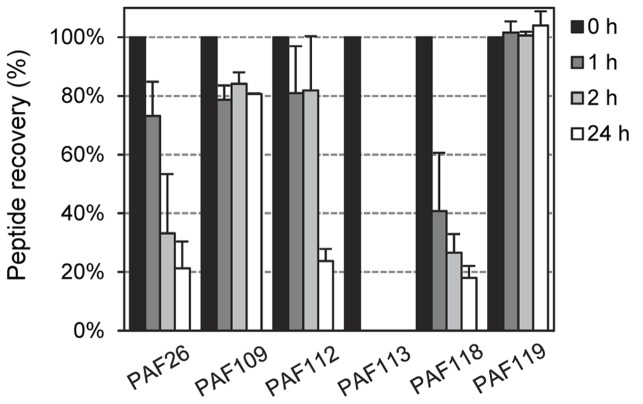
**Time-dependent peptide degradation in the presence of proteinase K.** Percentage of recovery of the input peptides (5 μM) represented as mean ± SD of two replicates, determined by the resulting chromatographic peak area of the corresponding peptide after treatment with proteinase K (5 μg/mL) for different times (0, 1, 2, and 24 h).

To exclude the possibility that increased sensitivity to proteolytic degradation could be the reason for limited antifungal activity, we tested the *in vitro* stability of the peptides derived from AfpB by incubation with proteinase K at different times and RP-HPLC analysis of the resultant digests. Again, we used peptide PAF26 as control as it is considered highly resistant to protease degradation (López-García et al., 2015). The RP-HPLC chromatograms (Supplementary Figure S3) showed that all the cyclic peptides derived from AfpB were, with varying degrees, resistant to protease treatment with a high dose of enzyme (5 μg/mL) (**Figure 8**). The antifungal peptides PAF112 and PAF118 show a degradation pattern similar to PAF26, with around 20% of peptide left after 24 h of treatment. Consistently among the different repetitions of this experiment, PAF118 showed faster degradation than PAF112. **Figure 8** shows a severe reduction of intact PAF118 after 1 and 2 h of treatment, which was different to the degradation of PAF112. Despite their similar antifungal activities and the presence of a disulfide bond in both sequences, PAF112 seems to be more resistant to protease degradation than PAF118. PAF113, which is a linear analog of PAF112, shows high sensitivity to proteinase K as it was completely degraded after only 1 h of treatment (**Figure 8**). We conclude that the presence of a disulfide bond increases PAF112 stability. Finally, the non-active peptides PAF109 and specially PAF119 were both remarkably resistant to degradation by proteinase K (**Figure 8**).

## Discussion

Most of the studies based on peptides derived from antifungal CRPs have focused on defensins and the highly conserved γ-motif (Sagaram et al., 2011; Avitabile et al., 2013; van der Weerden and Anderson, 2013; Muñoz et al., 2014; Kaewklom et al., 2016), but not on fungal AFPs. Our data identify for the first time synthetic peptides derived from fungal AFPs with antifungal activity. The peptides PAF112 and PAF118 derived from the *P. digitatum* AfpB and PAF116 from the *P. chrysogenum* PAF showed specific activity against selected filamentous fungi. PAF112 and PAF118 are not active against bacteria (*E. coli*) at the highest concentration tested (128 μM, **Figure 2**), which is consistent with the specificity profile of native AFPs that are antifungal but not antibacterial (Marx et al., 2008; Meyer, 2008). Importantly, none of the peptides overlap in neither of the two AFPs with the conserved γ-core motif that is present in virtually all known AFPs and other defensin-like antimicrobial peptides (Yount and Yeaman, 2004). There is no correlation between the GRAVY value and the activity of the peptides designed in our study, while all the active peptides are cationic (**Tables 1**, **2**). The importance of positive charge in the activity of AMPs including AFPs is well established (Brogden, 2005; Marcos et al., 2008). For example, three different mutations of the *P. chrysogenum* PAF in cationic lysine residues located at different positions resulted in loss of antifungal activity (Batta et al., 2009), and mutation of the arginine and/or lysine residues in the PAF26 sequence also abolished activity (Muñoz et al., 2013a). However, PAF112 and PAF116 show similar antifungal activity despite differences in charge (i.e., PAF116 is markedly more cationic). Therefore, the antifungal activity of these peptides can only be partially attributed to their positive net charge (see also below).

The *P. chrysogenum* PAF is the antifungal protein that has been most extensively characterized by site-directed mutagenesis, which allowed the identification of residues important for activity (Batta et al., 2009; Sonderegger et al., 2016, 2017). Both PAF112 and PAF116 are located in the analogous large loop L3 of the corresponding class B protein AfpB and class A protein PAF, respectively. Although these peptides share only limited sequence identity, they represent one of the regions with highest similarity between both proteins. Mutations of Lys35 and Lys38 (Batta et al., 2009) and of Phe31 (Sonderegger et al., 2016), which are located in this L3 loop of *P. chrysogenum* PAF, negatively affect antifungal activity. Therefore, by demonstrating the activity of isolated peptides from two different proteins (AfpB and PAF), our study contributes to establish L3 as an important domain in the antifungal activity of this class of AMPs. The peptide PAF112 is not structured (**Figure 7**), simulating the situation in the exposed loop L3 of the protein model, which lacks a defined canonical secondary structure (**Figures 1**, **2**). The structural data have shown that loop L3 is a dynamic and flexible region in PAF (Batta et al., 2009; Fizil et al., 2015).

The antifungal peptide PAF118 folds into an antiparallel β-sheet conformation that resembles the folding of this motif within the context of the protein. None of the previously reported mutations in residues of the PAF protein that affect the protein activity locate in the protein region analogous to PAF118 (Batta et al., 2009; Sonderegger et al., 2016). However, very recently it has been shown that the exchange of the negatively charged Asp19 to a neutral Ser in this PAF protein domain severely reduced protein activity (Sonderegger et al., 2017). This mutation does not disturb the overall 3D protein solution structure, but influences the surface charge distribution in distant regions of the PAF protein including the cationic L3, which most probably accounts for the loss of antifungal function (Sonderegger et al., 2017). The primary sequence of PAF and AfpB is different in this region as for instance no anionic residues are present in AfpB, contrary to PAF. Our demonstration of activity of the isolated motif represented by PAF118 identify this as a second putative region involved in the activity of AfpB. The only two cationic residues in this peptide are located in the turn that connects the two antiparallel β-sheets, and represent candidates to determine the antifungal properties of PAF118. Future work will attempt to confirm the importance of this domain in the activity of AFP-like proteins.

On the other hand, PAF109, which contains the minimal γ-core element, did not show either antifungal or antibacterial activity with any of the microorganism tested in our study. Consistent with the archetypical γ-core motif (Yount and Yeaman, 2004), this peptide sequence spans two antiparallel β-sheets (**Figure 2B**). Plant defensins are related to AFPs in that they are small and cationic CRPs that have antifungal activity and contain the γ-core motif; however, their αβ fold is structurally distinct from that of AFPs (van der Weerden and Anderson, 2013; Silva et al., 2014). Analogous γ-core peptides from the plant defensins MtDef4 from *Medicago*, SolyC07g007760 from tomato, or BhDef2 from *Brassica* were antimicrobial (Sagaram et al., 2011; Avitabile et al., 2013; Kaewklom et al., 2016). However, other γ-core peptides such as the two motifs derived from the *Medicago* defensin MsDef1 did not show antifungal activity against *Fusarium graminearum* (Sagaram et al., 2011). In previous studies, a correlation between the cationic net charge of the γ-core-derivative peptides and their antimicrobial activity was demonstrated. The peptide derivative BhDEF12M from BhDef1 protein contains asparagine to arginine substitutions that promoted antibacterial and antifungal activity (Kaewklom et al., 2016). Yet, there are exemptions to this rule as other non-cationic γ-core peptides derived from plant defensins (i.e., BhDef23) were antimicrobial (Kaewklom et al., 2016). In this context, PAF109 is a peptide that has low positive charge (ca. +1.1 at pH 7.0) and did not show activity. In addition, PAF109 and PAF119 are extremely stable peptides. Based on our data, we propose that the γ-core in AfpB has mainly a structural significance important for the formation of the native structure and the stability of the protein but is not necessarily associated with antimicrobial activity. However, the γ-core in the *P. chrysogenum* PAF has a higher cationic charge than the AfpB γ-core and mutation of Lys9 in the PAF protein resulted in the decrease of protein activity suggesting that this motif is important for protein function (Batta et al., 2009). Future investigations will address this apparently contradictive issue in fungal AFPs.

Antifungal activity of PAF116 is about 5–10 times weaker than the activity of the corresponding PAF protein. This finding implicates that additional motifs/sequences in the protein are required to achieve full activity. Similarly, we predict that PAF112 and PAF118 also have significantly lower activity than the elusive (non-detectable) AfpB. In order to test this hypothesis, our current efforts are directed toward the heterologous expression of the *afpB* gene to achieve protein production and purification. As an alternative approach in this study, we conducted experiments to determine whether treatment with both PAF112 and PAF118 inhibits fungal growth with potency higher than the sum of both peptides acting individually. We could demonstrate a positive synergistic interaction (**Figure 5**), which supports our hypothesis that both domains could cooperate to achieve the activity of the full-length protein.

It is generally established that antimicrobial compounds that have a positive synergistic interaction act on different microbial targets or have mechanisms of action that complement each other (Glattard et al., 2016; Vriens et al., 2016). This would be the case for the different AFP-derived and PAF26 peptides on *P. digitatum* and also for the PAF26/PAF protein combination on *A. niger* and *P. digitatum* as described in this study. Different time-course inhibition by PAF26 or AfpB-derived peptides on either *P. digitatum* or *A. niger* indicate mechanistic differences (**Figure 3**). The existence of at least partially different mechanisms in the combination PAF26/PAF protein is also supported by the different profile of activity among different fungal strains (**Table 3**). Furthermore, the demonstration of synergy also opens up new alternatives to improve (i) the antifungal activity of AFPs by combined action with other unrelated peptides such as PAF26, or (ii) the rational design of novel AMPs by combination of distinct peptide sequences (Badosa et al., 2009; Rodriguez Plaza et al., 2014).

Besides, there are also common mechanistic features between AFP proteins and the unrelated synthetic peptide PAF26, such as the endocytic internalization and increased concentrations of intracellular reactive oxygen species and cytoplasmic Ca^2+^ in sensitive fungal cells (Marx et al., 2008; Binder et al., 2010; Carmona et al., 2012; Muñoz et al., 2012). PAF26 has been proposed as a model peptide to study the mode of action of cell penetrating antifungal peptides (Muñoz et al., 2013a,b). The mechanism of PAF26 has been divided in three stages: (i) interaction with the fungal cell surface (cell wall and membrane), (ii) cellular internalization, and (iii) intracellular killing. This model framework could be useful to better refine and characterize the mechanism of AFPs and related proteins. Additionally, the identification of PAF112, PAF118, and PAF116 provides minimal motifs useful for the detailed mechanistic study of AFP-like proteins.

AFPs show remarkable thermal stability and resistance to protease degradation, which has been attributed to their tight structural packaging and disulfide bonding (Batta et al., 2009; Fizil et al., 2015). Consistently with this, all the AfpB-derived peptides with disulfide bonds were stable under restrictive *in vitro* degradation experiments (**Figure 8**). The stability of PAF109 and PAF119 indicates that their lack of antifungal activity cannot be attributed to propensity to degradation. By characterizing the linear analog PAF113 of peptide PAF112 we confirmed that the disulfide bridge between the two cysteines greatly stabilizes the peptide toward proteolytic degradation by proteinase K (Avitabile et al., 2013). PAF113 shows initial antifungal activity comparable to PAF112, although the activity was gradually lost with time in some experiments (data not shown), probably as consequence of degradation. The stability of all four cyclized AfpB-derived peptides suggests a reasonable stability of the putative protein AfpB. In that case, other so far unidentified regulatory mechanisms in mRNA stability and/or protein translation could be responsible for the lack of AfpB detection in *P. digitatum* cultures.

In summary, this study identifies protein motifs involved in the specific antifungal activity of AFPs, and opens new strategies for the future use of AFP-related peptides and proteins, the development of rationally designed antifungal peptides and their use in the characterization of the antifungal activity of AFPs. Two synthetic peptides that span about the central half of AfpB, including the major L3 loop, would contribute to specific antifungal activity of the putative AfpB, whereas the γ-core motif seems to play a different functional role.

## Author Contributions

SG, PM, and JM conceived and designed the study. SG modeled the AfpB structure. SG, MG, PM, and JM designed the peptides. SG, MG, and FM performed antimicrobial experiments. FM provided the PAF protein. AB performed the ECD experiments. SG and PM performed the degradation experiments. SG run statistical analyses. SG, PM, and JM wrote the paper. All authors approved the manuscript and agree to be accountable for the content of the work. Authorship is limited to those who have contributed substantially to the work reported.

## Conflict of Interest Statement

The authors declare that the research was conducted in the absence of any commercial or financial relationships that could be construed as a potential conflict of interest.

## Footnotes

1 http://swissmodel.expasy.org/

2 http://zhanglab.ccmb.med.umich.edu/ModRefiner/

3 http://mordred.bioc.cam.ac.uk/~rapper/rampage.php

4 http://web.expasy.org

5 http://www.cbs.dtu.dk/services/SignalP/

6 http://disulfind.dsi.unifi.it/

7 http://www.statgraphics.com/

## References

[B1] Arnold K. Bordoli L. Kopp J. Schwede T. (2006). The SWISS-MODEL workspace: a web-based environment for protein structure homology modelling. *Bioinformatics* 22 195–201. 10.1093/bioinformatics/bti770 16301204

[B2] Avitabile C. Capparelli R. Rigano M. M. Fulgione A. Barone A. Pedone C. (2013). Antimicrobial peptides from plants: stabilization of the γ core of a tomato defensin by intramolecular disulfide bond. *J. Pept. Sci.* 19 240–245. 10.1002/psc.2479 23420649

[B3] Badosa E. Ferré R. Francés J. Bardají E. Feliu L. Planas M. (2009). Sporicidal activity of synthetic antifungal undecapeptides and control of *Penicillium* rot of apples. *Appl. Environ. Microbiol.* 75 5563–5569. 10.1128/AEM.00711-09 19617390 PMC2737905

[B4] Batta G. Barna T. Gáspári Z. Sándor S. Kövér K. E. Binder U. (2009). Functional aspects of the solution structure and dynamics of PAF - A highly-stable antifungal protein from *Penicillium chrysogenum*. *FEBS J.* 276 2875–2890. 10.1111/j.1742-4658.2009.07011.x 19459942 PMC4290664

[B5] Binder U. Chu M. Read N. D. Marx F. (2010). The antifungal activity of the *Penicillium chrysogenum* protein PAF disrupts calcium homeostasis in *Neurospora crassa*. *Eukaryot. Cell* 9 1374–1382. 10.1128/EC.00050-10 20622001 PMC2937333

[B6] Brogden K. A. (2005). Antimicrobial peptides: pore formers or metabolic inhibitors in bacteria? *Nat. Rev. Microbiol.* 3 238–250. 10.1038/nrmicro1098 15703760

[B7] Brown G. D. Denning D. W. Gow N. A. R. Levitz S. M. Netea M. G. White T. C. (2012). Hidden killers: human fungal infections. *Sci. Transl. Med.* 4 165rv113. 10.1126/scitranslmed.3004404 23253612

[B8] Carmona L. Gandía M. López-García B. Marcos J. F. (2012). Sensitivity of *Saccharomyces cerevisiae* to the cell-penetrating antifungal peptide PAF26 correlates with endogenous nitric oxide (NO) production. *Biochem. Biophys. Res. Commun.* 417 56–61. 10.1016/j.bbrc.2011.11.050 22120633

[B9] Ceroni A. Passerini A. Vullo A. Frasconi P. (2006). Disulfind: a disulfide bonding state and cysteine connectivity prediction server. *Nuc. Acids Res.* 34 W177–W181. 10.1093/nar/gkl266 PMC1538823 16844986

[B10] Ferre R. Badosa E. Feliu L. Planas M. Montesinos E. Bardají E. (2006). Inhibition of plant-pathogenic bacteria by short synthetic cecropin A-melittin hybrid peptides. *Appl. Environ. Microbiol.* 72 3302–3308. 10.1128/AEM.72.5.3302-3308.2006 16672470 PMC1472336

[B11] Fisher M. C. Henk D. A. Briggs C. J. Brownstein J. S. Madoff L. C. McCraw S. L. (2012). Emerging fungal threats to animal, plant and ecosystem health. *Nature* 484 186–194. 10.1038/nature10947 22498624 PMC3821985

[B12] Fizil GÁ. áspári Z. Barna T. Marx F. Batta G. (2015). “Invisible” conformers of an antifungal disulfide protein revealed by constrained cold and heat unfolding, CEST-NMR experiments, and molecular dynamics calculations. *Chem. Eur. J.* 21 5136–5144. 10.1002/chem.201404879 25676351 PMC4464532

[B13] Fjell C. D. Hiss J. A. Hancock R. E. W. Schneider G. (2012). Designing antimicrobial peptides: form follows function. *Nat. Rev. Drug Discov.* 11 37–51. 10.1038/nrd3591 22173434

[B14] Galgóczy L. Kovács L. Vágvölgyi C. (2010). “Defensin-like antifungal proteins secreted by filamentous fungi,” in *Current Research, Technology and Education Topics in Applied and Microbial Biotechnology*, ed. Méndez-Vila A. (Badajoz: Formatex), 550–559.

[B15] Galgóczy L. Virágh M. Kovács L. Tóth B. Papp T. Vágvölgyi C. (2013). Antifungal peptides homologous to the *Penicillium chrysogenum* antifungal protein (PAF) are widespread among Fusaria. *Peptides* 39 131–137. 10.1016/j.peptides.2012.10.016 23174348

[B16] Garrigues S. Gandía M. Marcos J. F. (2016). Occurrence and function of fungal antifungal proteins: a case study of the citrus postharvest pathogen *Penicillium digitatum*. *Appl. Microbiol. Biotechnol.* 100 2243–2256. 10.1007/s00253-015-7110-3 26545756

[B17] Gasteiger E. Hoogland C. Gattiker A. Duvaud S. E. Wilkins M. R. Appel R. D. (2005). “Protein identification and analysis tools on the ExPASy server,” in *The Proteomics Protocols Handbook*, ed. Walker J. M. (Totowa, NJ: Humana Press), 571–607. 10.1385/1-59259-890-0:571

[B18] Glattard E. Salnikov E. S. Aisenbrey C. Bechinger B. (2016). Investigations of the synergistic enhancement of antimicrobial activity in mixtures of magainin 2 and PGLa. *Biophys. Chem.* 210 35–44. 10.1016/j.bpc.2015.06.002 26099623

[B19] Hancock R. E. W. Sahl H. G. (2006). Antimicrobial and host-defense peptides as new anti-infective therapeutic strategies. *Nat. Biotechnol.* 24 1551–1557. 10.1038/nbt1267 17160061

[B20] Kaewklom S. Euanorasetr J. Intra B. Panbangred W. Aunpad R. (2016). Antimicrobial activities of novel peptides derived from defensin genes of *Brassica* hybrid cv Pule. *Int. J. Pept. Res. Ther.* 22 93–100. 10.1007/s10989-015-9488-2

[B21] Kovács L. Virágh M. Takó M. Papp T. Vágvölgyi C. Galgóczy L. (2011). Isolation and characterization of *Neosartorya fischeri* antifungal protein (NFAP). *Peptides* 32 1724–1731. 10.1016/j.peptides.2011.06.022 21741420

[B22] López-García B. Harries E. Carmona L. Campos-Soriano L. López J. J. Manzanares P. (2015). Concatemerization increases the inhibitory activity of short, cell-penetrating, cationic and tryptophan-rich antifungal peptides. *Appl. Microbiol. Biotechnol.* 99 8011–8021. 10.1007/s00253-015-6541-1 25846331

[B23] López-García B. Pérez-Payá E. Marcos J. F. (2002). Identification of novel hexapeptides bioactive against phytopathogenic fungi through screening of a synthetic peptide combinatorial library. *Appl. Environ. Microbiol.* 68 2453–2460. 10.1128/AEM.68.5.2453-2460.2002 11976121 PMC127571

[B24] Lovell S. C. Davis I. W. Arendall Iii W. B. De Bakker P. I. W. Word J. M. Prisant M. G. (2003). Structure validation by Cα geometry: φ, ψ and Cβ deviation. *Proteins* 50 437–450. 10.1002/prot.10286 12557186

[B25] Marcos J. F. Gandía M. Harries E. Carmona L. Muñoz A. (2012). “Antifungal peptides: exploiting non-lytic mechanisms and cell penetration properties,” in *Small Wonders: Peptides for Disease Control* eds Rajasekaran K. Cary J. W. Jaynes J. Montesinos E. (Washington, DC: American Chemical Society), 337–357.

[B26] Marcos J. F. Muñoz A. Pérez-Payá E. Misra S. López-García B. (2008). Identification and rational design of novel antimicrobial peptides for plant protection. *Annu. Rev. Phytopathol.* 46 273–301. 10.1146/annurev.phyto.121307.094843 18439131

[B27] Marx F. Binder U. Leiter É. Pócsi I. (2008). The Penicillium chrysogenum antifungal protein PAF, a promising tool for the development of new antifungal therapies and fungal cell biology studies. *Cell. Mol. Life Sci.* 65 445–454. 10.1007/s00018-007-7364-8 17965829 PMC11131716

[B28] Meyer V. (2008). A small protein that fights fungi: AFP as a new promising antifungal agent of biotechnological value. *Appl. Microbiol. Biotechnol.* 78 17–28. 10.1007/s00253-007-1291-3 18066545

[B29] Muñoz A. Chu M. Marris P. I. Sagaram U. S. Kaur J. Shah D. M. (2014). Specific domains of plant defensins differentially disrupt colony initiation, cell fusion and calcium homeostasis in *Neurospora crassa*. *Mol. Microbiol.* 92 1357–1374. 10.1111/mmi.12634 24773060

[B30] Muñoz A. Gandía M. Harries E. Carmona L. Read N. D. Marcos J. F. (2013a). Understanding the mechanism of action of cell-penetrating antifungal peptides using the rationally designed hexapeptide PAF26 as a model. *Fungal Biol. Rev.* 26 146–155. 10.1016/j.fbr.2012.10.003

[B31] Muñoz A. Harries E. Contreras-Valenzuela A. Carmona L. Read N. D. Marcos J. F. (2013b). Two functional motifs define the interaction, internalization and toxicity of the cell-penetrating antifungal peptide PAF26 on fungal cells. *PLoS ONE* 8:e54813 10.1371/journal.pone.0054813 PMC3549957 23349973

[B32] Muñoz A. Marcos J. F. Read N. D. (2012). Concentration-dependent mechanisms of cell penetration and killing by the *de novo* designed antifungal hexapeptide PAF26. *Mol. Microbiol.* 85 89–106. 10.1111/j.1365-2958.2012.08091.x 22646057

[B33] Palicz Z. Jenes T. Gáll T. Miszti-Blasius K. Kollár S. Kovács I. (2013). In vivo application of a small molecular weight antifungal protein of *Penicillium chrysogenum* (PAF). *Toxicol. Appl. Pharmacol.* 269 8–16. 10.1016/j.taap.2013.02.014 23466426

[B34] Petersen T. N. Brunak S. Von Heijne G. Nielsen H. (2011). SignalP 4.0: Discriminating signal peptides from transmembrane regions. *Nat. Methods* 8 785–786. 10.1038/nmeth.1701 21959131

[B35] Pettersen E. F. Goddard T. D. Huang C. C. Couch G. S. Greenblatt D. M. Meng E. C. (2004). UCSF Chimera - A visualization system for exploratory research and analysis. *J. Comput. Chem.* 25 1605–1612. 10.1002/jcc.20084 15264254

[B36] Rodriguez Plaza J. G. Morales-Nava R. Diener C. Schreiber G. Gonzalez Z. D. Ortiz M. T. L. (2014). Cell penetrating peptides and cationic antibacterial peptides: two sides of the same coin. *J. Biol. Chem.* 289 14448–14457. 10.1074/jbc.M113.515023 24706763 PMC4031501

[B37] Sagaram U. S. Pandurangi R. Kaur J. Smith T. J. Shah D. M. (2011). Structure-activity determinants in antifungal plant defensins MsDef1 and MtDef4 with different modes of action against *Fusarium graminearum*. *PLoS ONE* 6:e18550 10.1371/journal.pone.0018550 PMC3076432 21533249

[B38] Shah D. M. Read N. D. (2013). Antifungal peptides come of age. *Fungal Biol. Rev.* 26 107–108. 10.1016/j.fbr.2013.01.001

[B39] Silva P. M. Gonçalves S. Santos N. C. (2014). Defensins: antifungal lessons from eukaryotes. *Front. Microbiol.* 5:97 10.3389/fmicb.2014.00097 PMC3960590 24688483

[B40] Sonderegger C. Fizil Á. Burtscher L. Hajdu D. Muñoz A. Gáspári Z. (2017). D19S Mutation of the cationic, cysteine-rich protein PAF: novel insights into its structural dynamics, thermal unfolding and antifungal function. *PLoS ONE* 12:e0169920 10.1371/journal.pone.0169920 PMC5224997 28072824

[B41] Sonderegger C. Galgóczy L. Garrigues S. Fizil Á. Borics A. Manzanares P. (2016). A *Penicillium chrysogenum*-based expression system for the production of small, cysteine-rich antifungal proteins for structural and functional analyses. *Microb. Cell Fact.* 15 192 10.1186/s12934-016-0586-4 PMC5106836 27835989

[B42] Tóth L. Kele Z. Borics A. Nagy L. G. Váradi G. Virágh M. (2016). NFAP2, a novel cysteine-rich anti-yeast protein from *Neosartorya fischeri* NRRL 181: isolation and characterization. *AMB Expr.* 6 75 10.1186/s13568-016-0250-8 PMC5025423 27637945

[B43] van der Weerden N. L. Anderson M. A. (2013). Plant defensins: common fold, multiple functions. *Fungal Biol. Rev.* 26 121–131. 10.1016/j.fbr.2012.08.004

[B44] Váradi G. Tõth G. K. Kele Z. Galgõczy L. Fizil A. Batta G. (2013). Synthesis of PAF, an antifungal protein from *P. chrysogenum*, by native chemical ligation: native disulfide pattern and fold obtained upon oxidative refolding. *Chem. Eur. J.* 19 12684–12692. 10.1002/chem.201301098 24175344

[B45] Virágh M. Vörös D. Kele Z. Kovács L. Fizil Á Lakatos G. (2014). Production of a defensin-like antifungal protein NFAP from *Neosartorya fischeri* in *Pichia pastoris* and its antifungal activity against filamentous fungal isolates from human infections. *Protein Expr. Purif.* 94 79–84. 10.1016/j.pep.2013.11.003 24269762

[B46] Vriens K. Cools T. L. Harvey P. J. Craik D. J. Braem A. Vleugels J. (2016). The radish defensins RsAFP1 and RsAFP2 act synergistically with caspofungin against *Candida albicans* biofilms. *Peptides* 75 71–79. 10.1016/j.peptides.2015.11.001 26592804

[B47] Xu D. Zhang Y. (2011). Improving the physical realism and structural accuracy of protein models by a two-step atomic-level energy minimization. *Biophys. J.* 101 2525–2534. 10.1016/j.bpj.2011.10.024 22098752 PMC3218324

[B48] Yount N. Y. Yeaman M. R. (2004). Multidimensional signatures in antimicrobial peptides. *Proc. Natl. Acad. Sci. U.S.A.* 101 7363–7368. 10.1073/pnas.0401567101 15118082 PMC409924

[B49] Zasloff M. (2002). Antimicrobial peptides of multicellular organisms. *Nature* 415 389–395. 10.1038/415389a 11807545

