# Supplementary material for: Mapping and Identification of Antifungal Peptides in the Putative Antifungal Protein AfpB from the Filamentous Fungus Penicillium digitatum
Source: Front Microbiol. 2017 Apr 6;8:592. doi: 10.3389/fmicb.2017.00592 (PMC5382200; doi:10.3389/fmicb.2017.00592)
Supplement: Supplementary file 1 [file Data_Sheet_1.DOCX]

Supplementary Material

**Mapping and identification of antifungal peptides in the putative antifungal protein AfpB from the filamentous fungus *Penicillium digitatum***

**Sandra Garrigues ^1^, Mónica Gandía ^1^, Attila Borics ^2^, Florentine Marx ^3^, Paloma Manzanares ^1^ and Jose F. Marcos ^2,^***

*** Correspondence:** Jose F. Marcos (jmarcos@iata.csic.es)

# Supplementary Figure S1


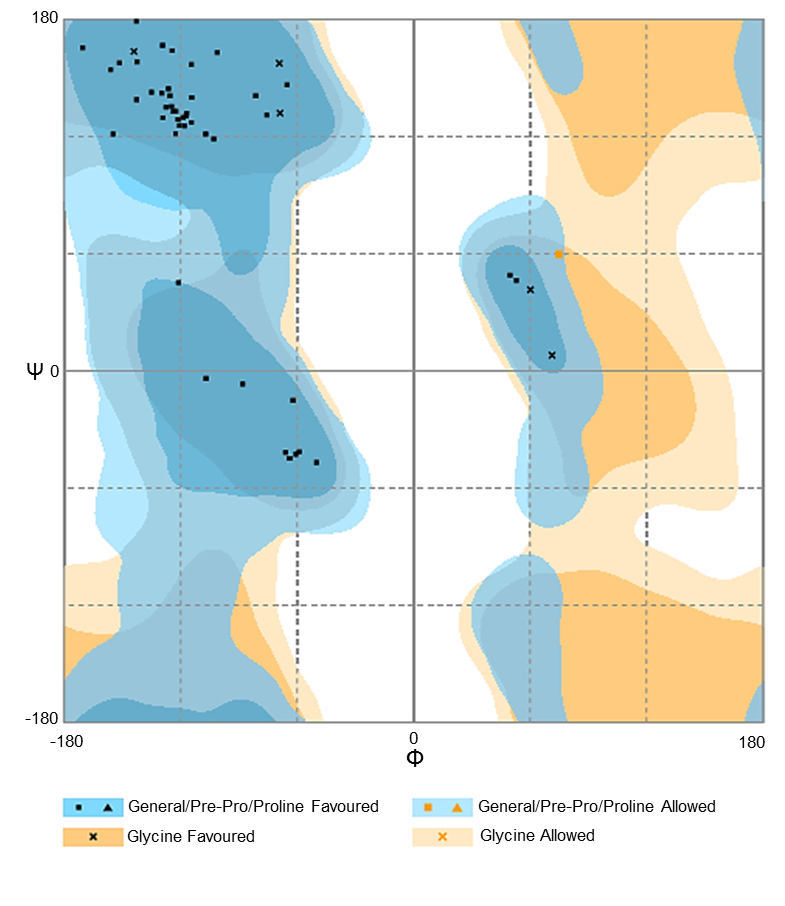


**Supplementary Figure S1.** **Ramachandran Plot of the structural model of AfpB.** Visualization of the energetically allowed regions for backbone dihedral angles ψ against φ of the amino acid residues in AfpB protein using RAMPAGE software tool. General amino acids are represented with a square. Proline residues are represented with a triangle. Glycine residues are represented with a cross. Energetically favored and allowed regions for all amino acids (excluding Glycine) are represented in dark and light blue respectively. Energetically favored and allowed regions for Glycine are represented in dark and light orange respectively. Amino acids located in energetically favored regions are colored in black. Amino acids located in energetically allowed regions are highlighted in orange. In AfpB refined molecular model, 56 amino acids out of 57 (98%) are located in energetically favored regions, while only 1 amino acid (2%) is located in energetically allowed regions.
