# Supplementary material for: Mapping and Identification of Antifungal Peptides in the Putative Antifungal Protein AfpB from the Filamentous Fungus Penicillium digitatum
Source: Front Microbiol. 2017 Apr 6;8:592. doi: 10.3389/fmicb.2017.00592 (PMC5382200; doi:10.3389/fmicb.2017.00592)
Supplement: Supplementary file 3 [file Data_Sheet_3.DOCX]

Supplementary Material

**Mapping and identification of antifungal peptides in the putative antifungal protein AfpB from the filamentous fungus *Penicillium digitatum***

**Sandra Garrigues ^1^, Mónica Gandía ^1^, Attila Borics ^2^, Florentine Marx ^3^, Paloma Manzanares ^1^ and Jose F. Marcos ^2,^***

*** Correspondence:** Jose F. Marcos (jmarcos@iata.csic.es)

# Supplementary Figure S3


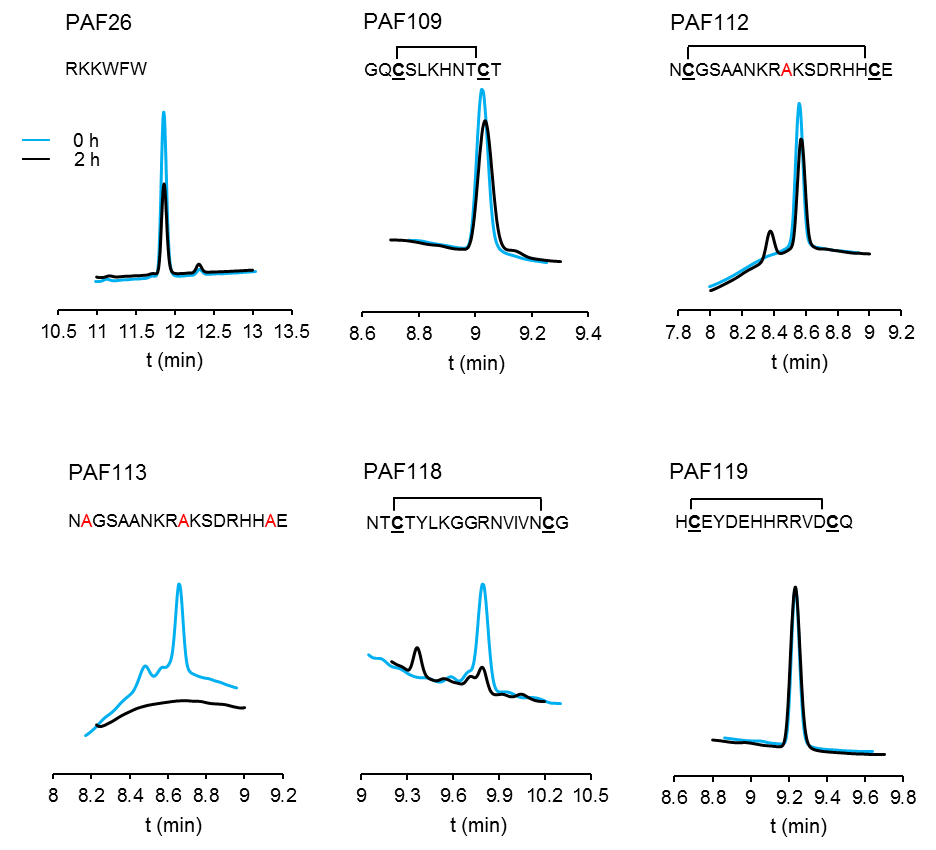


**Supplementary Figure S3.** **RP-HPLC chromatograms of the AfpB-derived peptides after treatment with proteinase K**. Superposition of representative RP-HPLC chromatograms of the corresponding peptides after treatment with proteinase K (5 µg/mL) for different times (0 and 2 h, blue and black, respectively). Retention times (min) are shown at the bottom. Black lines on the peptides sequences show the disulfide bonds in the AfpB-derived PAF109, PAF112, PAF118 and PAF119. Cysteine residues forming disulfide bonds are represented in bold and underlined.
